# Progress in Disease of Digestive Organs

**Published:** 1901-06-15

**Authors:** 


### Progress in Disease of Digestive Organs.

**Stomach.**—Oral sepsis as a frequent cause of disease is discussed at length by W. Hunter.[1] The organisms due to decaying teeth are peculiarly virulent, and being continually produced lead more often than is supposed not only to chronic gastric catarrh, but also to various septic diseases. The stomach is unable to destroy them except just at the full tide of digestion; the chronic catarrh lessens even this power, and then the mucous membrane itself becomes a new culture ground for them. In some cases after removal of one or two suppurating stumps or fixed plates all the gastric symptoms, the ashy grey complexion, and general depression quickly disappear. Among other results of oral sepsis he finds cases of fever, hæmorrhages, septicæmia, pernicious anæmia, ulcerative endocarditis, and toxic neuritis. Thus well-marked cases of multiple neuritis with tingling, paralyses, wasting of muscles, and loss of knee-jerk have been found existing with chronic dental suppuration, and quickly recover when it is removed. Stomatitis, whether simple or ulcerative, tonsillitis, and pharyngitis are often put down to all sorts of causes except the source of toxins present in decayed teeth, and surgical operations are too often undertaken while the mouth remains a sink of infection, perhaps causing no local pain, but producing the most virulent type of organisms, viz., those grown in diseased bone.

**Membranous Œsophagitis.**—Nathan Raw[2] records the vomiting of a complete cast of the œsophagus by a man who had been in the habit of drinking neat whiskey, as many persons in the north of Great Britain do. He regards it as an instance of phlegmonous inflammation of the connective tissue, and can only find five similar cases. There had been some dysphagia for six months, and lately the patient had taken only warm liquids on account of the pain. A soft stomach tube passed with difficulty, but his condition remained unaltered. Finally violent coughing came on, and a cast $8\frac{1}{2}$ inches long, tough, and elastic, composed of the inner layers of the œsophagus as far as the muscularis externa, was brought up, together with much pus. The patient afterwards became unable to drink on account of the pain, and gastrotomy was performed, but life was not prolonged more than six weeks.

**Coated Tongue.**—A. L. Gillespie[3] remarks how little importance is given to the condition of the tongue to-day as a diagnostic sign, whereas it was formerly one of the chief, if not actually the primary, means of diagnosis. He refers to the work of Müller and Fuchs, who found that in health a much larger percentage of old people have clean tongues than young and middle-aged persons have. In acute diseases there is much more coating than in chronic. Indeed in chronic gastritis the tongue is more often clean than in health. Whenever less food is taken, and therefore less mechanical attrition takes place, fur collects, especially if the person has abnormally long papillæ. Still, in some diseases a true desquamative catarrh occurs, and here masses of epithelial cells are more abundant than in health.

**Dilatation of the Œsophagus** may be due to obstruction or (rarely) of the idiopathic type. Max Einhorn[4] notices among the symptoms dysphagia and a feeling of pressure in the chest, becoming very severe at times, with regurgitation of food. If a tube is introduced 12 inches the contents of the dilatation return through it, and a photo should be taken by X-rays after a solution of bismuth has been introduced. We must make a diagnosis from malignant strictures, diverticula, and small unimportant sacculations.

**Air Swallowing** was noticed in these columns in an account of a paper by G. A. Sutherland some time ago. Mathieu and Follet[5] hold that it is often confounded with flatulent dyspepsia. A feeling of weight in the stomach after meals may give colour to this. Thirty or forty, or even hundreds of eructations of odourless gas take place. Pressure on certain spots, such as the epigastrium, will bring on an attack, and in severe cases extreme vomiting and emaciation may result. If the larynx is watched a movement of deglutition will be seen before each eructation. These neurotic patients have usually acquired the habit during slight real dyspepsia.

**Dilatation of the Stomach** is said to be caused in some cases by pressure of the superior mesenteric vessels and nerve on the third part of the duodenum. Byron Robinson[6] operated on such a case recently, and has noted 20 others in which the dilatation corresponded to this line. D. D'Esterre reports[7] a death from tetanic convulsions following chronic dilatation. The patient learnt to wash out his stomach, and the symptoms of dilatation were much relieved. Painful convulsions, with once some opisthotonos and risus sardonicus, came on, from which he recovered under sedative treatment. A few months later a similar but fatal attack followed. The writer is inclined to accept the theory of poisoning from the absorption of decomposed matters. Combemale and Huríez[8] report epileptic attacks in like circumstances. The attacks were at first of the type of *petit mal*, but later on exactly resembled the major form. They only occurred when the vomiting, which he suffered from, had ceased. It is suggested that ordinary epilepsy may be due to auto-intoxication of this kind. L. Bouveret, of Lyons,[9] gives a useful sign of the earlier stages of pyloric stenosis, which may precede or alternate with the appearance of waves of contraction from the cardiac to the pyloric end. In these patients the left half of the stomach is found to be rigid and more prominent than the right. This tonic spasm vanishes from time to time, but does not travel from place to place, and would seem to be a useful aid to diagnosis at a time when there is little anorexia, and neither vomiting nor a tumour are present. Rolleston[10] finds that some 45 cases of congenital hypertrophy of the pylorus with stenosis have been recorded, and gives a photograph of the large tumour in his own patient which could not be felt during life. Of the six cases operated on only one recovered, but there are six not included in the list which are believed to have recovered without operation. This patient suffered from vomiting and convulsions when 14 days old, then diarrhœa and vomiting; the latter recurred again and again with constipation and convulsions, probably from toxic absorption. Death took place at the end of the second month. In spite of obstinate vomiting and constipation, it does not seem possible to make a complete diagnosis if a tumour cannot be discovered. The question is raised whether some of the slighter cases which do not prove fatal may not in after life be met with among sufferers from simple pyloric stenosis. Rectal feeding may be of temporary use, and nasal feeding may minimise the spasmodic stenosis and allow nutrition to be carried on in mild degrees of the affection.

**Hyperchloridria.**—Riegel[11] shows that morphia, however given, increases the secretion of HCl, and he therefore thinks it injurious in this affection, especially when associated with an ulcer. The suggestion has been made that atropine should be combined with morphia in these cases. Luigi[12] believes that continuous hypersecretion is generally due to retention of food from pyloric obstruction; occasionally, when this obstruction is spasmodic, the cause may be a hyperæsthesia of the stomach or minute ulcerations. He would not deny the possibility of a hypersecretion without stasis, but has never found a case in thousands of examinations. W. Russell[13] thinks that simple hyperchloridria is more common than is supposed. In typical cases there is pain one to three hours after a meal, distension, heartburn, acid eructations, and sometimes vomiting. The symptoms are relieved by taking a glass of any bland fluid, such as milk, or another meal. At times there is merely indefinite discomfort. The cause of the symptoms is the presence of free HCl, whether there is an actual increase of the free acid or a hypersensitiveness of the stomach. Two methods of treatment have been adopted by different schools. It is clear that salivary digestion is cut short because an excess of starch is found in these stomachs; hence some give a proteid diet to combine with the acid, and to reduce the amount of undigested starch; others actually give a carbohydrate diet to cause the production of less acid. Fat seems to have a favourable influence, and the proteid diet certainly gives speedy relief. The writer thinks the choice of diet depends on the severity of the case and on the use of easily digested and unirritating foods of either kind.

**Gastric Ulcer.**—J. J. Walsh[14] refers to the ulcers which present a pure culture of the pneumococcus, and to those found as a complication of appendicitis as forms which are not often recognised. Olive oil in daily doses of 3 to 10 oz. has been found useful for ulcer, though its mode of action is not clear. The action of the gastric juice cannot be, according to Einhorn, the sole causal factor, for ulcers have been found where HCl was entirely absent; still they are often associated with hyperacidity. Hæmorrhagic erosions should be distinguished from ulcer. There is often subacidity, the pain is not localised, comes on about half an hour after food, and is of a burning but not crampy character. The patients, says C. Parises, are afraid to eat, and become emaciated. A more liberal diet may be often given than in ulcer, and lavage with nitrate of silver is beneficial. This affection is by some thought to be a form of chronic gastritis. Hernia of the linea alba may also be confounded with ulcer. A. F. Hornberg[15] reports 30 cases due to traumatism, emaciation, and hernial tendencies. Thus digestive disorders coming on after a fall, or pain after muscular efforts, disappearing when the horizontal posture is assumed, should lead to the investigation of the site of these herniæ. In 22 cases operated on 12 were cured and two had relapses.

[1] Practit., Dec. [2] Lancet, Jan. 5. [3] Edin. Med. Jour., Jan. [4] Practit., Feb. [5] Lancet, Mar. 30. [6] Practit., Feb., p. 219. [7] Lancet, Dec. 22. [8] Treatment, Nov. [9] La Semaine Med., Ap. 3. [10] Practit., Feb., and Brit. Med. Jour., Dec. 22. [11] Treatment, Nov. [12] Med. Rec., Oct. 27. [13] Scot. Med. Jour., Feb. [14] Med. Rec., Dec. 5. [15] Med. Rec., Dec. 29.

